# High-Throughput Non-Contact Vitrification of Cell-Laden Droplets Based on Cell Printing

**DOI:** 10.1038/srep17928

**Published:** 2015-12-14

**Authors:** Meng Shi, Kai Ling, Kar Wey Yong, Yuhui Li, Shangsheng Feng, Xiaohui Zhang, Belinda Pingguan-Murphy, Tian Jian Lu, Feng Xu

**Affiliations:** 1 School of Energy and Power Engineering, Xi’an Jiaotong University, Xi’an, 710049, P.R. China; 2 Bioinspired Engineering and Biomechanics Center (BEBC), Xi’an Jiaotong University, Xi’an, 710049, P.R. China; 3 State Key Laboratory of Mechanical Structure Strength and Vibration, School of Aerospace, Xi’an Jiaotong University, Xi’an, 710049, P.R. China; 4 MOE Key Laboratory of Biomedical Information Engineering, School of Life Science and Technology, Xi’an Jiaotong University, Xi’an, 710049, P.R. China; 5 Department of Biomedical Engineering, Faculty of Engineering, University of Malaya, Lembah Pantai, 50603 Kuala Lumpur, Malaysia

## Abstract

Cryopreservation is the most promising way for long-term storage of biological samples *e.g.*, single cells and cellular structures. Among various cryopreservation methods, vitrification is advantageous by employing high cooling rate to avoid the formation of harmful ice crystals in cells. Most existing vitrification methods adopt direct contact of cells with liquid nitrogen to obtain high cooling rates, which however causes the potential contamination and difficult cell collection. To address these limitations, we developed a non-contact vitrification device based on an ultra-thin freezing film to achieve high cooling/warming rate and avoid direct contact between cells and liquid nitrogen. A high-throughput cell printer was employed to rapidly generate uniform cell-laden microdroplets into the device, where the microdroplets were hung on one side of the film and then vitrified by pouring the liquid nitrogen onto the other side via boiling heat transfer. Through theoretical and experimental studies on vitrification processes, we demonstrated that our device offers a high cooling/warming rate for vitrification of the NIH 3T3 cells and human adipose-derived stem cells (hASCs) with maintained cell viability and differentiation potential. This non-contact vitrification device provides a novel and effective way to cryopreserve cells at high throughput and avoid the contamination and collection problems.

Cryopreservation has been widely used for long-term preservation of cells, aggregates, tissues and organs. Careful cryopreservation maintains the functional properties (*e.g.*, proliferation and differentiation) and genetic characteristics of cellular constructs, and protects them from infection, allowing them to be available for off-the-shelf use in both research and clinical applications^1^,^2^. As for now, a variety of cryopreservation methods have been developed including slow freezing and vitrification^3^,^4^,^5^, among which vitrification offers several advantages over slow freezing in terms of maintaining viability, genetic profiles and cytoskeletal structure of cells^6^,^7^. Thus, vitrification holds great promise for applications in biomedical fields such as reproduction, tissue engineering, and organ transplantation to achieve better cryopreservation outcome^8^,^9^,^10^.

The advantages of vitrification attribute to its high cooling rate to transform cell suspensions with cryoprotectant (CPA) from the aqueous phase to a glass state directly^11^, which can avoid the cryoinjury induced by intracellular ice formation during freezing process^12^,^13^. Besides, the high cooling rate can allow using low CPA concentrations in freezing, thereby reducing the potential CPA toxicity and osmotic damage of cells^5^,^14^. Various methods have been developed to increase cooling rate, such as open pulled straw (OPS)^15^, quartz micro capillary (QMC)^14^, cryotop^16^, and droplet-based vitrification^5^,^17^,^18^. For instance, in a droplet-based vitrification method, cell-laden droplets with sizes down to nanoliter were ejected (*e.g.*, by cell printer) directly into liquid nitrogen at high throughput (~0.14 nL per droplet, 1,000 droplets/s^5^). Due to the small volume of the droplets, a significant high cooling rate was achieved at a relatively low CPA concentration (*e.g.*, 4.5% ectoine^17^), which can potentially improve the efficiency of freezing and reduce the risk of toxicity from high CPA concentrations^5^,^18^. With this method, a variety of cells, such as red blood cells^17^, hepatocytes cells^5^, oocytes^19^ and even whole blood^20^ have been successfully preserved. However, these approaches are associated with several limitations. Firstly, most methods (*i.e.*, OPS, QMC, cryotop and droplet-based vitrification) have a high contamination risk with pathogenic agents due to a direct contact of the cells with non-sterile liquid nitrogen^21^,^22^. For instance, bone marrow has been found to be contaminated by hepatitis virus in liquid nitrogen during cryopreservation, which caused patient deaths after transplantation^23^. Although the vapour phase storage system was developed to avoid the bio-pollutions in liquid nitrogen during storage^24^,^25^, it is still challenging to avoid the potential contamination between bio-specimens and liquid nitrogen during freezing and thawing processes. Although “straw in straw” method has been employed to reduce the risk, its cooling rate was significantly decreased due to increased thermal barrier (~400 °C/min)^26^. Secondly, cryotop and droplet-based methods involve the potential issue of sample loss due to an inefficient cell collection. For example, during droplet-based vitrification, the liquid nitrogen forms nitrogen vapour layer around the droplet that pushes droplets to move around due to Leidenfrost phenomenon, thus making it difficult for an efficient cell collection^27^. Additionally, the liquid nitrogen vapour blanket significantly decreases the cooling rate of droplets^27^,^28^. Although precooled substrates have been used to avoid the Leidenfrost phenomenon during freezing process^18^, the temperature of substrate (~193 K) is not low enough (at least below glass transition temperature of the droplet, *i.e.*, <141 K) to achieve high cooling rate and this approach still has the contamination problem while storing in liquid nitrogen. Therefore, there is still an unmet need to develop an effective method to vitrify cells without a direct contact with liquid nitrogen but with a high cooling rate and convenient collection means.

Herein, we developed a novel device that enables cell vitrification without a direct contact with liquid nitrogen. With this device, cells can be vitrified at a high cooling rate on the surface of an ultra-thin and highly thermal conductive silver film via heat transfer from liquid nitrogen boiling on the other side of the freezing film. High warming rate can also be achieved by immersing the device in a water bath at 37 °C. Furthermore, a cell printer ejected the cell suspension as well-controlled droplets array at high throughput onto the vitrification device before vitrification. We investigated the freezing ability of the vitrification device through the theoretical and experimental analysis of spatiotemporal variations of temperature and crystallization. In addition, we experimentally and theoretically determined the size effect of cell-laden droplets (*i.e.*, NIH 3T3 cells and hASCs) on cell viability and functionalities such as differentiation potential. Our findings suggest that this novel vitrification device can be used to vitrify cells at high throughput with low risk of contamination and cell loss.

## Materials and Methods

### Device Design

**Figure 1:**
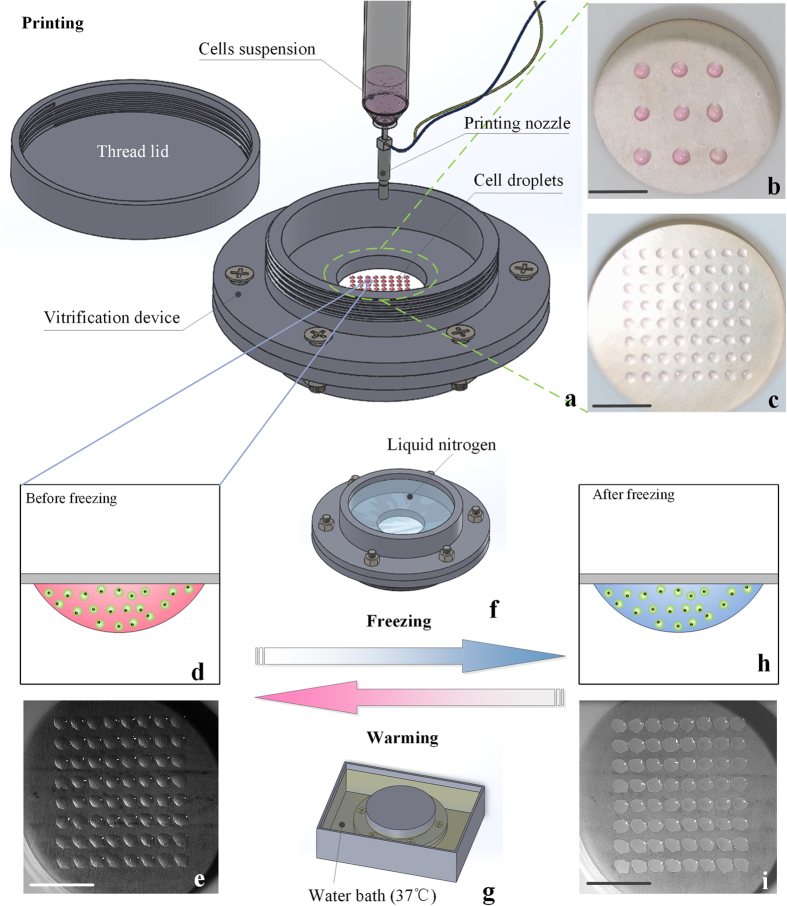
The cell printing based non-contact vitrification device. (**a**) Droplets arrayed by cell printer; (**b,c**) Various of droplet patterns; (**d,e**) Droplets before freezing; (**f,g**) Freezing and warming methods; (**h,i**) Frozen droplets. Scale bars: 10 mm.

The device consisted of four components. There are two chambers (a *liquid nitrogen chamber* and a *cell chamber*) separated by a 150 μm silver film (Fig. 1a), and a thread lid used to seal the *cell chamber*. A silver film was used as the freezing film due to its high thermal conductivity (429 W/(m•K)^29^), biocompatibility and antibacterial ability^30^. The two chambers and thread lid were made of Polytetrafluoroethylene (PTFE) to prevent the device from potential chemical corrosion and deformation during the freezing process. Before attaching a freezing film between the chambers, a thin layer of low temperature sealant (DG-4, China Bluestar Cheng Rand Chemical Co.) was spread on the exposed surfaces of the two chambers to prevent any leakage of the liquid nitrogen from the *liquid nitrogen chamber*. Then the two chambers were fastened together by using plastic bolts.

The *cell chamber* was used to hold the cell-laden droplets, which were patterned on the freezing film by a custom-developed cell printer (Fig. 1a) before vitrification. Due to the high throughput and precise spatial controllability of the cell printer, the cell-laden droplets could be rapidly ejected on freezing film with various volumes and arrays (Fig. 1b,c). To protect cell-laden droplets from potential contamination in air, the thread lid (Fig. 1a) was developed to seal the *cell chamber*. The *liquid nitrogen chamber* was designed to allow pouring of liquid nitrogen into device for freezing (Fig. 1f), and allow the warm water getting into device for rewarming (Fig. 1g). Due to the existence of the silver film, the cell-laden droplets could be separated with liquid nitrogen/warm water, thus the contamination problem could be avoided. As all the cell-laden droplets could be maintained on the freezing film during and after freezing/thawing (Fig. 1e,i), the cells can be 100% collected. Meanwhile, the device could be stored in liquid nitrogen after freezing, where liquid nitrogen stayed on the freezing film without direct contact with the droplets.

### Theoretical Analysis

For a better understanding of the freezing phenomenon, we developed a theoretical model to simulate the vitrification process on the vitrification device. The vitrification can be divided into two coupled processes, *i.e.* the heat transfer process and the crystallization process^27^. Accordingly, two coupled equations were used to describe the heat transfer and crystallization processes.

The crystallization equation^31^:


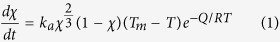


**Figure 2:**
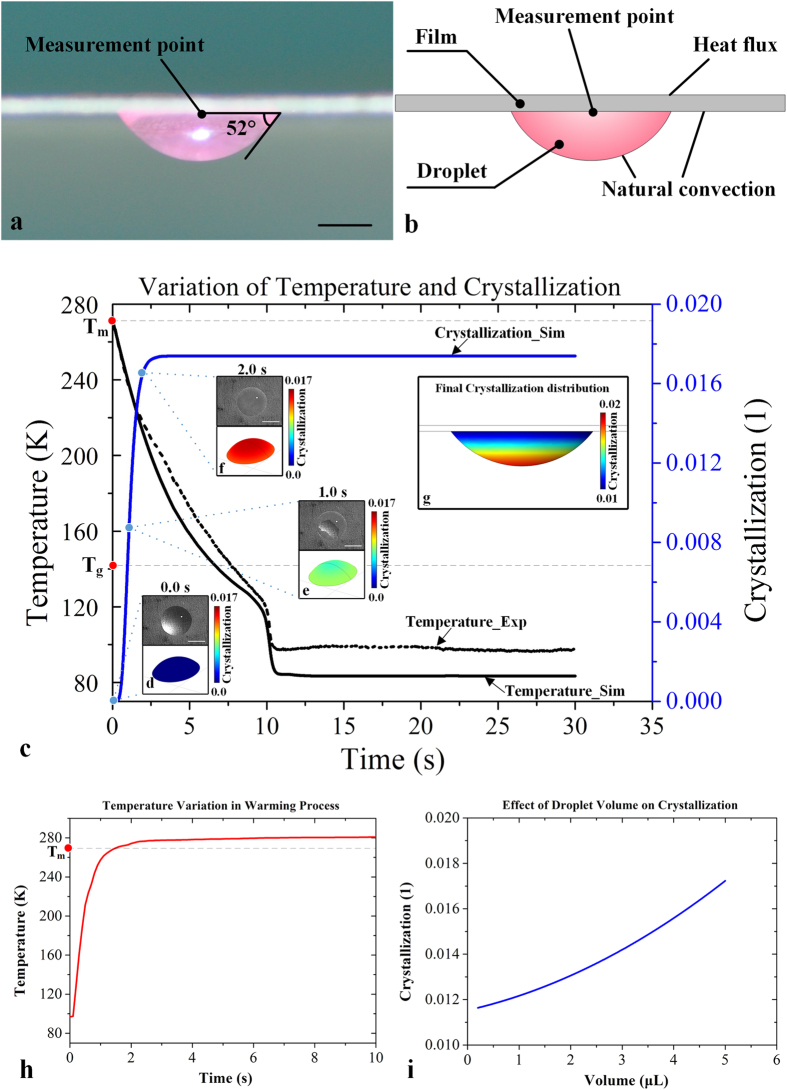
Experimental and numerical analysis of droplets frozen in non-contact device. (**a**) Morphology of droplet hanging on the freezing film; (**b**) Schematic diagram shows geometric model and boundary conditions of a droplet; (**c**) Experimental and numerical results of temperature and crystallization variation of a droplet during freezing; (**d–f**) Crystallization in different time points (*upper: recorded by high speed camera (scale bar: 1* *mm); lower: numerical results*); (**g**) Final crystallization distribution in a droplet; (**h**) Experimental temperature variation in warming process; (**i**) Crystallization prediction in different droplets.

The heat transfer equation:


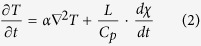


where *χ* represents the degree of crystallization (0 < *χ* < 1), *t* (s) is time. *T* (K) is the temperature of the droplet, and *T*_*m*_ (K) is the temperature at the end of melting. *Q* (J/mol) is the activation energy, *R* (J/mol·K) is the gas constant, and *k*_*a*_ is the characteristic coefficient of the crystallization. *α* (m^2^/s) is the thermal diffusivity, where *α = λ/ρc*_*p*_. *ρ* (kg/m^3^), *c*_*p*_ (kJ/(kg•K)) are the density and the specific heat at constant pressure, respectively, and *λ* (W/(m•K)) is the thermal conductivity. *L* (J/kg) is the latent heat. The boundary condition of heat flux was applied on the surface of the film exposing to the liquid nitrogen^32^,^33^, and the nature convection was assumed for other boundary exposing to the air^34^ (Fig. 2b). The detail ofthe coupled equations and boundary conditions were illustrated in Supplementary Information. These equations were solved by finite element method in COMSOL software.

For an accurate and authentic theoretical model, the morphological parameters (*e.g.*, contact angle) of the droplets hanging on the freezing film were experimentally measured and used to build the geometric models in SOLIDWORKS^®^. Due to the surface tension, the droplet hung on the film with a contact angle of 52° and formed an approximate spherical cap shape (Fig. 2a). Moreover, other geometric parameters were calculated and shown in Supplementary Information (Fig. S1).

### Experimental Design

#### Heat transfer experiments

To evaluate the freezing ability (*i.e.*, cooling rate) of our vitrification device, the temperature variation of vitrification device was measured during liquid nitrogen boiling. The thermocouple (T type, Omega) was located on the bottom of a 5 μL droplet that was hung on the downside of freezing film to measure the temperature variation (Fig. 2a). 5 μL droplet was used for analysis because it was a commonly used droplet volume in vitrification^35^,^36^,^37^. Further, 5 μL droplet was sufficient to cover the thermocouple (~0.4 mm) for measurements. To verify the theoretical model, these experimental data were used to compare with calculated results of the same position (Fig. 2b). To evaluate the warming rate of this vitrification device, we also measured the temperature variation of warming process (37 °C water bath) by a thermocouple located at the same position of the freezing film.

#### Visualization of freezing process

To observe of the freezing process, a high-speed camera (Olympus®, i-Speed TR 4GB MONO) was utilized to record the freezing process of droplets hanging on the freezing film. The variation of a single 5 μL droplet and 8 × 8 array of droplets were recorded to compare with numerical results. In order to record the bottom view of the droplets and to observe the crystallization process, an experimental apparatus was built up to arrange the lens of high-speed camera under the device.

#### Cell culture

To investigate the ability of the non-contact device in preserving cell viability and functionalities after freezing, the device was employed to vitrify mouse embryonic fibroblast cells (NIH 3T3 cells) and hASCs. NIH 3T3 cells were cultured in DMEM with high glucose (Corning Cellgro, Manassas, USA) supplemented with 10% (v/v) fetal bovine serum (FBS) and 1% (v/v) antibiotic/antimycotic (Gibco, New York, USA) under 5% CO_2_ at 37 °C. On the other hand, hASCs were isolated using methods as described in a previous study^38^. hASCs were cultured in F12/DMEM consisted of 10% (v/v) FBS and 1% (v/v) antibiotic/antimycotic under 5% CO_2_ at 37 °C. hASCs were sub-cultured to passage 3 prior to vitrification.

#### Cell printing and vitrification

To evaluate the freezing ability of our device, the NIH 3T3 cells or hASCs were frozen by this method. First, NIH 3T3 cells (1 × 10^6^ cells/mL) or hASCs (1 × 10^5^ cells/mL, passage 3) were suspended in a cryomedium containing 10% (v/v) DMSO in DMEM. Cell suspensions were then printed on the freezing film (the cell chamber side) into 8 × 8 array using the custom-designed cell printer^39^ (Fig. 1a,e). The pneumatic pressure of cell printer was set at 0.03 MPa. By tuning the electromagnetic valve opening duration to 5000 μs, 1000 μs and 200 μs, we obtained 5 μL, 1 μL and 0.2 μL per droplet respectively. To avoid the contamination brought by frequent usage of the device, a strict sterilization (*i.e.*, ultraviolet and alcohol sterilization) was applied and the printing nozzle of cell printer (for printing cells droplets) was changed each time upon vitrification of different types of cells.

After ejecting droplets and sealing the device with a thread lid, the device was turned over with the *liquid nitrogen chamber* upward, allowing the cell-laden droplets to hang on the film by surface tension (Fig. 1d,e). To vitrify the cell-laden droplets, liquid nitrogen were poured into the *liquid nitrogen chamber* (Fig. 1f). The droplets were rapidly frozen via boiling heat transfer of liquid nitrogen and heat conduction from the film to droplets (Fig. 1h,i). During warming, the whole device was put into 37 °C water bath with the *cell chamber* upward (Fig. 1g), which allowed warm water to get into the *liquid nitrogen chamber* and directly contact with the surface of freezing film. Thus, the cell-laden droplets on the other surface of freezing film could be warmed quickly via heat conduction. As the *cell chamber* was sealed by thread lid, the cell droplets would not contact with the warm water, thus the contamination could be avoided. After thawing, the cell-laden droplets were suspended with cell culture medium followed by centrifugation to remove the CPA. NIH 3T3 cells or hASCs were resuspended with complete medium and then subjected to cell viability evaluation. In addition, hASCs were cultured for evaluation of morphology and osteogenic potential.

#### Evaluation of cell viability

Live-dead staining was used to evaluate the cell viability. Both NIH 3T3 cells and hASCs recovered from vitrification/thawing process were incubated in live-dead staining solution (calcein-AM/EB, Molecular Probes, Eugene, OR) at 37 °C for 30 mins. The images of live and dead cells were captured using a fluorescent microscope (Olympus, IX-81, Tokyo, Japan). The total number of live and dead cells was counted using ImagePro Plus 6.0 software (Media cybernetics. Inc., Bethesda, MD) and the cell viability was calculated as the ratio of live cells to total number of cells.

#### Osteogenic differentiation assay

To induce osteogenic differentiation, vitrified and fresh hASCs (non-vitrified hASCs at passage 3) were cultured in osteogenic induction medium composed of complete culture medium, 100 nM dexamethasone (Sigma), 10 mM b-glycerophosphate (Sigma), and 0.5 mM ascorbic acid-2-phosphate (Sigma) for 10 days. After 10 days, differentiation of hASCs into osteogenic-like cells was assessed by calcium deposition stained using Alizarin Red (Sigma). Calcium deposition was quantified using ImagePro Plus 6.0 software and expressed as % of the stained area.

#### Statistical analysis

All statistical analysis were carried out using a SPSS 18.0 software. One-Way ANOVA with tukey post hoc test was employed to compare data of cell viability among various droplet sizes. Data of osteogenic potential in fresh and vitrified hASCs were compared using an independent *t-test*. Each datum was expressed as mean ± standard error of mean (SEM). Statistical significance was accepted at *p* < 0.05. All experimental protocols were carried out in accordance with the guidelines, as approved by Xi’an Jiaotong University.

## Results

### Numerical simulation analysis

In this study, we developed a novel device for non-contact vitrification of cells (Fig. 1). To understand the freezing phenomenon in the vitrification device, we numerically simulated the freezing process that occurred on the device. The variations of temperature and crystallization in a 5 μL droplet were acquired via numerical simulation (Fig. 2c, temperature: red curve; crystallization: blue curve). We observed that the temperature decreased dramatically after freezing started, and reached the glass transition temperature (for 10% DMSO: T_g_ = 141 K^40^) at t = 6.7 s. In vitrification, the temperature region from melting temperature (for 10% DMSO: T_m_ = 269 K^41^) to glass transition temperature is called the dangerous temperature region (DTR)^27^. Thus, the cooling rate of our device passing the DTR is ~1146 K/min, which is higher than the reported cooling rate of several vitrification methods in previous studies^26^,^42^,^43^. For example, human embryo was effectively vitrified with 400 K/min with “straw in straw method”^26^. Besides, the cooling rate of our device is beyond the critical cooling rate of many kinds of CPA^44^,^45^,^46^, such as VS55 (~2.5K /min), DP6 (~40 K/min) and 35% 1,2-propanediol (294 K/min), indicating that our device has a good compatibility with these CPAs. At the same time, the crystallization increased rapidly at initial stage during freezing and reached stable state soon. The simulation results indicate that the crystallization process of droplet started immediately after the liquid nitrogen was poured into device and completed in around 2 seconds, as confirmed by the recorded video (Fig. 2d–f). It is worth mentioning that, in the initial stage of freezing, there was a moving boundary of crystallization from the bottom (close to freezing film) to the top (far from freezing film) of droplet surface (Supplementary Information, Movie S1). Thus it induced a low crystallization area at the time of 1 s, which can be seen both from the high-speed camera record (Fig. 2e, upper picture, the transparent area in the center) and simulation result (Fig. 2e, lower picture, the blue area at top). However, after freezing process completed, we observed a nonhomogeneous crystallization levels, which increased from the bottom to the top of droplet (Fig. 2g), which is due to the relatively higher cooling rate at the droplet bottom (closer to the film) compared to that at droplet top. It indicated that if the cells within a droplet can be located near the film surface during freezing (*e.g.*, culturing cells on freezing film until adherent before freezing), the crystallization of cells may be decreased and the cell viability may be improved. Moreover, to predict the effect of droplet size on the effectiveness of vitrification device, we carried out simulation to evaluate the crystallization using droplets with volumes in the range of 0.2 to 5 μL, which were commonly applied for vitrification of embryos^35^,^47^, oocytes^48^ and mammalian cells^4^,^49^. We observed that the crystallization decreased with decreasing droplet volume (Fig. 2i), which is straightforward to understand.

### Heat transfer experimental results

To evaluate the freezing ability (*i.e.*, cooling rate) of our vitrification device, we measured and analysed the variation of temperature distribution in the droplet during the freezing process. The temperature variation was obtained by thermal experiments (Fig. 2c, black dot line), which agreed well with the simulation data (Fig. 2c, black solid line). In particular, we observed a sharp temperature decrease when it reached around 120 K at t = 10 s from both the experimental and simulated results (Fig. 2c, black dot and solid line). That is mainly because the liquid nitrogen boiling got into the transition boiling region and the cooling heat flux was gradually increased to critical heat flux (CHF)^32^,^33^, which results in a high cooling heat flux and therefore a high cooling rate of droplets. The heat flux variation of the pool boiling of liquid nitrogen was shown and discussed in Supplementary Information (Fig. S2). Meanwhile, there was a temperature difference of ~15 K between numerical and experimental results, which might be attributed to the environmental heating and large heat capacity of the PTFE chambers in experimental circumstance. However, this temperature difference did not affect the variation of temperature between DTR (*i.e.*, 269K-141K) which was significant for crystallization formation (Fig. 2c, black dot line: experiment; black solid line: simulation). In warming process, we observed that temperature of droplet sharply increased from 97 K to melting temperature (for 10% DMSO: T_m_ = 269 K^41^) in ~1.5 s (Fig. 2h). Thus, the warming rate of our device was ~6880 K/min, which was comparable to and even beyond other vitrification experiments (*e.g.*, the warming rate was 3280 K/min for mouse oocytes in 0.25 mL open straw)^50^. It indicates that our vitrification device has enough ability to rewarm cell-laden droplets avoiding the harmful recrystallization in the warming process.

### Survival rate of vitrified NIH 3T3 cells

**Figure 3:**
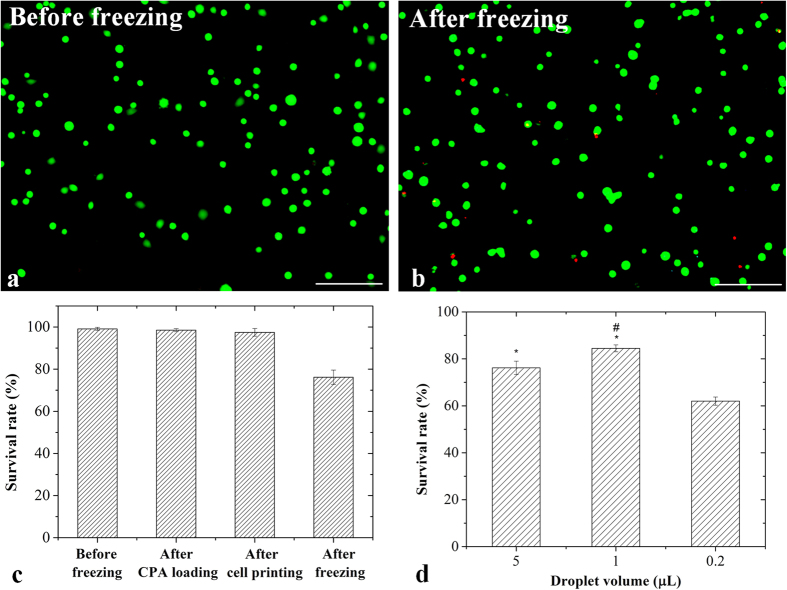
The NIH 3T3 cells droplets frozen on non-contact vitrification device. Representative fluorescent images of cells before (**a**) and after freezing (**b**) (green: live cells; red: dead cells; scale bar: 100 μm); (**c**) Survival rate at different processes; (**d**) Survival rate of cells frozen in various droplet volumes. *indicates *p < 0.05* relative to 0.2 ul droplet; ^#^indicates *p < 0.05* relative to 5 ul droplet.

To examine the efficiency of our device on maintaining cell viability after vitrification, we performed vitrification experiments on NIH 3T3 cells using our non-contact device (Fig. 3). From the results of live/dead cell staining, we observed that most of the cells remained alive after freezing (Fig. 3a,b). Then we quantified cell viability and found that there was no significant difference (*p* > 0.05) in cell viability before CPA loading (99 ± 1%), after CPA loading (98 ± 1%) and printing (97 ± 2%) (Fig. 3c), indicating that cell viability was not affected by CPA loading and cell printing. At the same time, we achieved 76% of cell viability of NIH 3T3 by freezing cells encapsulated in 5 μL droplets using our device, indicating the ability of our device in maintaining high cell viability of NIH 3T3 cells. To improve the cell viability of NIH 3T3, we tried to vitrify cells in smaller droplets. It was observed that the survival rate of NIH 3T3 cells was improved to 84 ± 2% (Fig. 3d) when 1 μL droplets were employed. These results were consistent with the fact that the cooling rate increases with a decreased droplet volume, which results in decreased risk of ice formation. However, when we further decreased the droplets volume to 0.2 μL, the survival rate of cells was sharply decreased to 62 ± 2% (Fig. 3d). This could be due to the evaporation of droplets that occurred throughout the cell printing and freezing process. The evaporation resulted in a rapid decrease of droplet volume and thus increase of CPA concentration. It caused more osmotic damage and chemical toxicity to the cells. In the time period from cell printing to freezing (~2 minutes), the 0.2 μL droplet significantly decreased to about 40% of its initial volume by evaporation, but 1 μL and 5 μL droplet remained about 80% and 90% of initial volume, respectively. Therefore, the evaporation occurred through this period and cannot be ignored in small droplets due to its high specific surface area. The further analysis of droplets evaporation was shown in Supplementary Information (Fig. S3).

### Survival rate and osteogenic potential of vitrified hASCs

**Figure 4:**
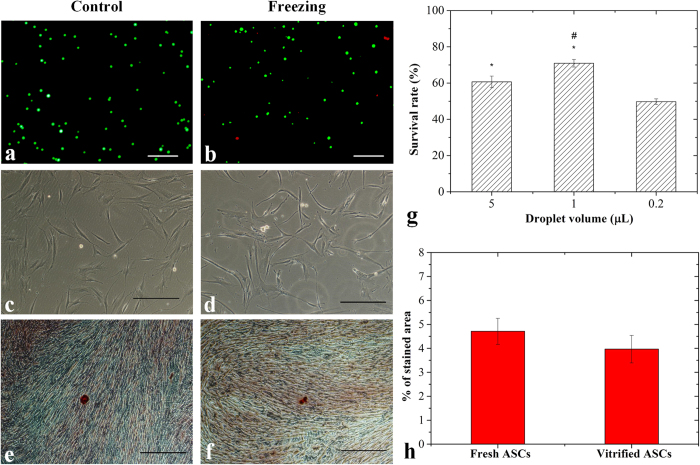
The hASCs droplets frozen on non-contact vitrification device. *Before freezing:* (**a**) Representative fluorescent images of hASCs; (**c**) Undifferentiated cells; (**e**) Differentiated cells; *After freezing:* (**b**) Representative fluorescent images of hASCs (green: live cells; red: dead cells); (**d**) Undifferentiated cells; (**f**) Differentiated cells; (**g**) Survival rate of hASCs frozen in various droplet volumes; (**h**) Alizarin Red stained area analysis. (Scale bar: 100 μm). ^*^indicates *p < 0.05* relative to 0.2 ul droplet; ^#^indicates *p < 0.05* relative to 5 ul droplet.

To evaluate the ability of our device in maintaining morphology and functional property (*e.g.*, osteogenic potential) of stem cells, we performed vitrification of hASCs using our device. First, we checked cell morphology of hASCs recovered from vitrification using an inverted light microscope. We observed that both vitrified and fresh hASCs (non-vitrified hASCs at passage 3) presented adherent and fibroblast-like shapes (Fig. 4c,d), which met the minimal criteria of characteristics of human mesenchymal stem cells^51^,^52^. To assess the osteogenic potential of the vitrified hASCs, we performed osteogenic induction on hASCs cultured for 3 days after recovery from vitrification. After 10 days of osteogenic induction, both the vitrified and fresh ASCs displayed the formation of calcium deposits as reflected by positive staining of Alizarin Red (Fig. 4e,f). Similar levels of calcium deposition were observed for the vitrified hASCs to those of fresh hASCs, indicating that the osteogenic potential of hASCs was not significantly affected by the vitrification using our device (Fig. 4h). Taken together, vitrification performed using our device maintained the morphology and osteogenic potential of hASCs. We further conducted live-dead staining assay to evaluate the viability of hASCs after vitrification. We found that cell viability of hASCs vitrified in 1 μl droplets (71 ± 2%) was significantly higher (*p* < 0.05) than those in 5 μl droplet (61 ± 4%). When we decreased the droplets volume to 0.2 μL, the cell viability of vitrified hASCs was significantly decreased (*p* < 0.05) (Fig. 4g), which might be due to evaporation of droplets.

## Discussion

In this study, we developed a novel non-contact vitrification device where the vitrification of cell-laden droplets was achieved through pool boiling of liquid nitrogen and heat transfer of an ultra-thin silver film. Although the freezing and warming rates offered by this vitrification device might not be higher than some of the existing opened vitrification systems (*e.g.*, OPS, QMC, cryotop and droplet-based vitrification)^4^, its cooling rate (~1146 K/min) and warming rate (~6880 K/min) were high enough to vitrify and rewarm cells with maintained cell viability and functional properties (*e.g.*, differentiation potential). More importantly, it had demonstrate its potential in addressing the issues of contamination and sample loss. It is worth mentioning that the frozen droplets were able to stay hanging on the film during and after the freezing/thawing process, mainly due to the hydrophilic film surface (θ < 90°)^53^ which leads to strong adhesion of ice^54^. This specific feature makes it convenient to collect the frozen droplets, as reflected by the 100% collection rate of this device. Even if some droplets might detach after freezing, the cells would still stay inside the chamber and would give a 100% recovery. Besides, this device refrained the Leidenfrost phenomenon between droplets and liquid nitrogen, which was regarded as a thermal barrier in droplet vitrification^28^,^55^. We observed that the silver film was oxidised after repeated uses, however, the oxidation layer did not have significant effect on the freezing/warming rate as confirmed with the thermal experiments in this study, and it was biocompatible to the cells^56^.

Another way to improve cooling rate and decrease crystallization was to reduce the droplet volume. However, droplets in extremely small size did not get a better performance due to the issue of evaporation. The reason was that the decrease of relative volume of droplet (*V* /*V*_0_) in evaporation mainly depended on the specific surface area of droplet (*S*_0_/*V*_0_) (Supplementary Information, equation (S-19))^57^,^58^. Therefore, a smaller droplet has higher evaporation ability due to its larger specific surface area. However, the evaporation equation also inspires us to develop a humidity-controlled environment of the experimental system to decrease pressure difference (Δ*P*), which could be a potential method to decrease evaporation, and the evaporation will be investigated in the further study.

From another view, larger droplets provides the better ability to vitrify large biospecimens, such as oocytes^50^, embryos^35^, cell aggregates^7^ and even tissues^59^, than smaller droplets (*e.g.*, 0.2 μL) as used in existing methods. In this sense, our device could be a potential non-contact vitrification method to freeze these large biospecimens since it had achieved successful cryopreservation in larger droplets (*i.e.*, 1 μL). Besides, the surface of freezing film could be modified by nanostructures to enhance the boiling heat transfer between liquid nitrogen and film^60^, and the cooling rate of our device could thus be further improved to freeze larger biospecimens more efficiently.

In conclusion, we developed a novel non-contact droplet-based vitrification device to address the contamination and collection issues existing in most vitrification settings to date, while integrating with cell printing technology to achieve high throughput cryopreservation. Both the experimental and numerical results indicated that, the non-contact vitrification device can offer a high cooling rate and high warming rate as well as to achieve successful vitrification and thawing of cell-laden droplets. Our findings demonstrated that this device has a great ability to maintain high cell viabilities and preserves differentiation potential of hASCs. Our non-contact device provides a novel solution for high-throughput, contamination-free and cell loss-free vitrification cryopreservation of cells with maintained cell viability and functionality. Due to its high cooling and warming rate, the device also can be a potential approach for the non-contact vitrification of large biomaterials, such as embryos, cell aggregates and even tissues.

## Additional Information

**How to cite this article**: Shi, M. *et al.* High-Throughput Non-Contact Vitrification of Cell-Laden Droplets Based on Cell Printing. *Sci. Rep.* **5**, 17928; doi: 10.1038/srep17928 (2015).

## Supplementary Material

Supplementary Movie S1

Supplementary Information
