# Supplementary material for: High-Throughput Non-Contact Vitrification of Cell-Laden Droplets Based on Cell Printing
Source: Sci Rep. 2015 Dec 14;5:17928. doi: 10.1038/srep17928 (PMC4677291; doi:10.1038/srep17928)
Supplement: Supplementary Information [file srep17928-s2.pdf]

Title:

High-Throughput Non-Contact Vitrification of Cell-Laden Droplets Based on Cell Printing

Authors:

Meng Shi [a,b], Kai Ling [b,c], Kar Wey Yong [b,e], Yuhui Li [b,d], Shangsheng Feng [b,c], Xiaohui Zhang [b,d], Belinda Pingguan-Murphy [e], Tian Jian Lu [b, c#], Feng Xu [b,d#]

[a] *School of Energy and Power Engineering, Xi'an Jiaotong University, Xi'an, 710049, P.R. China*

[b] *Bioinspired Engineering and Biomechanics Center (BEBC), Xi'an Jiaotong University, Xi'an, 710049, P.R. China*

[c] *State Key Laboratory of Mechanical Structure Strength and Vibration, School of Aerospace, Xi'an Jiaotong University, Xi'an, 710049, P.R. China*

[d] *MOE Key Laboratory of Biomedical Information Engineering, School of Life Science and Technology, Xi'an Jiaotong University, Xi'an, 710049, P.R. China*

[e] *Department of Biomedical Engineering, Faculty of Engineering, University of Malaya, Lembah Pantai, 50603 Kuala Lumpur, Malaysia*

[#] *Corresponding authors*

Contact information

Tian Jian Lu (Corresponding author): tjlu@mail.xjtu.edu.cn

Feng Xu (Corresponding author): fengxu@mail.xjtu.edu.cn

Supplementary Information

1. Geometric parameters of different hanging droplets

To improve the accuracy of simulation, the contact angle of hanging droplet was measured and the geometric parameters were calculated before building geometric models (**Fig. S1**) in SOLIDWORKS® and importing to COMSOL®.

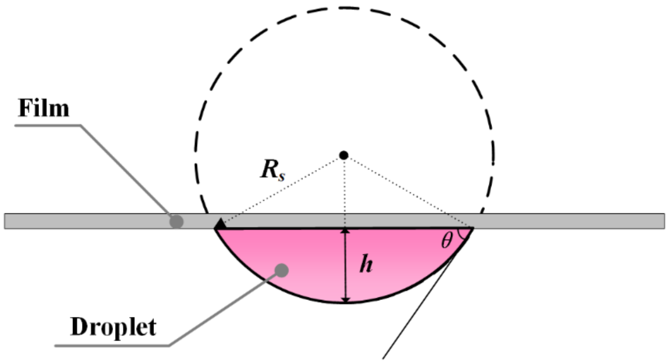

Figure S1 The geometric sketch of hanging droplet on freezing film

The volume of spherical cap shaped droplet is:

$$V = \frac{\pi R_s^3}{3}\beta \tag{S-1}$$

Where the V is volume of droplet, R_s is the radius of spherical droplet and β is the geometric parameters related with contact angle:

$$\beta = (1 - \cos\theta)^2(2 + \cos\theta) \tag{S-2}$$

Thus, if we know the volume and contact angle of spherical cap shaped droplet on the freezing film, the radius of spherical droplet will be:

$$R_s = (\frac{3V}{\pi\beta})^{\frac{1}{3}} = (\frac{3V}{\pi(1-\cos\theta)^2(2+\cos\theta)})^{\frac{1}{3}} \quad \text{(S-3)}$$

The height of spherical cap shaped droplet will be:

$$h = R_s(1-\cos\theta) \quad \text{(S-4)}$$

The contact radius of spherical cap shaped droplet will be:

$$r = R_s \sin\theta \quad \text{(S-5)}$$

And the surface area of spherical cap shaped droplet can be obtained:

$$S = 2\pi R_s^2(1-\cos\theta) \quad \text{(S-6)}$$

The geometric parameters of different hanging droplets used in simulation were shown below:

Table S1 Geometric parameters of different hanging droplets

Volume (V)	Diameter(R_s)	Height (h)	Contact radius (r)	Specific surface area (S_0/V_0)
5 μl	2.30 mm	0.89 mm	1.82 mm	257529 m^{-1}
1 μl	1.35 mm	0.52 mm	1.06 mm	440368 m^{-1}
0.2 μl	0.79 mm	0.30 mm	0.62 mm	753018 m^{-1}

Where, S_0 and V_0 represent the initial surface area and volume of spherical cap shaped droplet respectively.

2. The control equations of vitrification

The crystallization equation. It is now well accepted that a lower degree of crystallization implies a higher efficiency of vitrification thereby a higher cell survival

rate[1]. The following non-isothermal kinetic equation proposed by Boutron and Mehl[2] is adopted to describe the crystallization process:

$$\frac{d\chi}{dt} = k_a \chi^{\frac{2}{3}} (1-\chi)(T_m - T) e^{-Q/RT} \tag{S-7}$$

The heat transfer equation. Transient heat conduction equation with a source term is adopted here to describe the heat transfer process during vitrification:

$$\frac{\partial T}{\partial t} = \alpha \nabla^2 T + \frac{\dot{\Phi}}{\rho c_p} \tag{S-8}$$

where $\dot{\Phi}$ (J/(m^3 s)) is the volumetric heat source as induced by the latent heat releasing upon freezing. The heat source $\dot{\Phi}$ is related to the degree of crystallization as:

$$\Phi = L \cdot \rho \cdot \chi \tag{S-9}$$

$$\dot{\Phi} = \frac{d\Phi}{dt} = L \cdot \rho \cdot \frac{d\chi}{dt} \tag{S-10}$$

where L (J/kg) is the latent heat. By substituting equation (S-10) into equation (S-8), the final heat transfer equation is obtained as:

$$\frac{\partial T}{\partial t} = \alpha \nabla^2 T + \frac{L}{c_p} \cdot \frac{d\chi}{dt} \tag{S-11}$$

Through this model, the spatial and temporal evolution of temperature and crystallization in the cell-laden droplets can be analyzed precisely to reveal the important characteristics of the device, such as cooling rate, the degree of crystallization, and final distribution of crystallization. Further, the required volume of droplets for an effective vitrification was calculated.

3. The boundary condition of the freezing film exposing to the liquid nitrogen

When liquid nitrogen was boiling on the substrate of the *liquid nitrogen chamber*, the heat flux at the surface of freezing film depended on the temperature difference between liquid nitrogen and substrate[3]. With the decrease of temperature of substrate, the pooling boiling of liquid nitrogen would experience three regions, *i.e.*, the film boiling, transition boiling and nucleate boiling[4]. The heat fluxes in these regions were different and would vary with temperature difference:

$$q = h\Delta T = h(T - T_{\mathrm{LN}}) \tag{S-12}$$

Where q is the heat flux. T and T_{LN} is the temperature of substrate and liquid nitrogen respectively. The h is the heat transfer coefficient and is illustrated by the equations below[3]:

$$\begin{cases} h = 125 + 0.069\times\Delta T, & 52 \le \Delta T \le 214 \\ h = 13097.8 - 723.04\times\Delta T + 13.48\times\Delta T^2 - 0.084\times\Delta T^3, & 19.6 \le \Delta T \le 52 \\ h = 82.74 - 131.22\times\Delta T + 37.64\times\Delta T^2 - 1.13\times\Delta T^3, & 4 \le \Delta T \le 19.6 \\ h = 21.945\times\Delta T, & 0 \le \Delta T \le 4 \end{cases} \tag{S-13}$$

From the equation (S-12) and equation (S-13), the heat flux of of liquid nitrogen boiling can be obtained and shown in **Figure S2**, and this heat flux was used as the thermal boundary condition in our simulation (**Fig. 2b**). When boiling began, due to the high temperature of substrate and large temperature difference between liquid nitrogen and substrate, the boiling state was film boiling and the heat flux was approximately linear variation with temperature of substrate. When the temperature decreased about 120 K, the film boiling started to transform into nucleate boiling, and the heat flux was dramatically increasing to critical heat flux (CHF) in transition

boiling. This rapidly increased heat flux resulted in sharply decreased temperature of freezing film whose data were shown in our article (**Fig. 2c**). When the temperature of substrate closed to liquid nitrogen temperature, the heat flux would decrease due to the limited temperature difference and induced the slow freezing rate in this region.

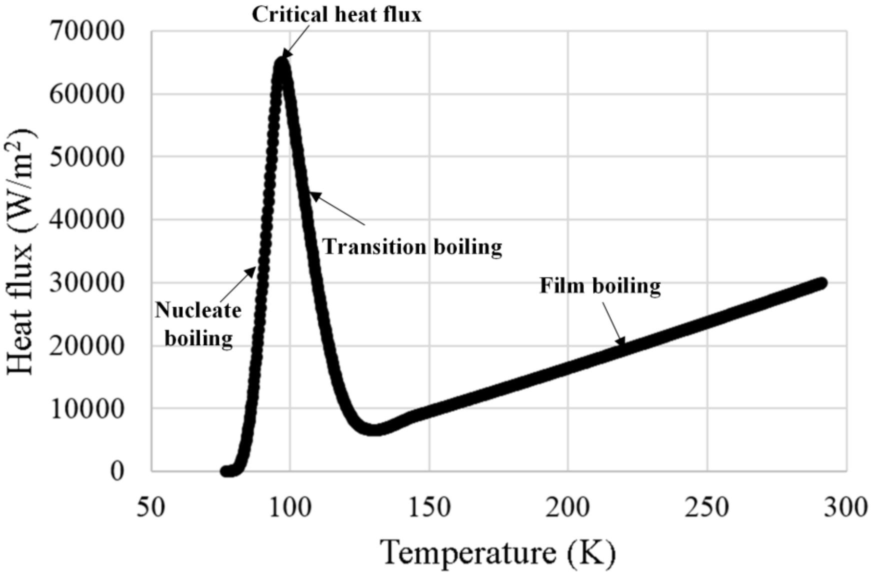

Figure S2 Variation of heat flux of liquid nitrogen boiling with the temperature of substrate

4. Analysis of droplet evaporation

Due to evaporation was an important factor in our experiment, the evaporation phenomenon of droplets hanging on the freezing film was experimental and theoretical analyzed.

Experimental analysis

We used a digital camera (D90, NIKON®) to record the morphological variation of droplets after printing on freezing film, and the results were shown in **Figure**

S3a. We observed that there was a distinct volume decrease in 0.2 μL droplets at 2 minutes after printing on the freezing film, while there were inconspicuous change in 1 μL and 5 μL droplets. It indicated that, during the time region from cell printing to freezing (about 2 minutes), the evaporation of small droplets (0.2 μL) was heavy and induced the cells exposing in high osmotic pressure and toxicity then caused the death of cells.

Theoretical analysis

Evaporation of sessile droplet cap can be illustrated as[5]:

$$\frac{dV}{dt} = -2\pi D\Delta P \frac{M}{\rho RT}(\frac{3V}{\pi\beta})^{\frac{1}{3}} f(\theta) \qquad \text{(S-14)}$$

Where, the V is the present volume of droplet, V_0 is the initial volume of droplet; D is the diffusivity of vapor molecular in the air. $\Delta P = P_0 - P_\infty$ is the is the pressure difference between the saturation vapor pressure of droplet (P_0) and the vapor pressure far away from the drop surface (P_∞). M is its molar mass, ρ is the density of the liquid, R is gas constant and T is temperature of droplet, respectively. The characteristic parameter θ is the contact angle of droplet on film surface, and $f(\theta)$ is a function of contact angle which considering the shape factors on evaporation:

$$f(\theta) = \begin{cases} 0.00008957 + 0.6333\theta + 0.116\theta^2 - 0.08878\theta^3 + 0.01033\theta^4, \theta > 10° \\ 0.6366\theta + 0.09591\theta^2 - 0.06144\theta^3, \qquad \theta < 10° \end{cases} \qquad \text{(S-15)}$$

Changing the form of equation (S-14):

$$\frac{dV}{V^{\frac{1}{3}}} = -2\pi D\Delta P \frac{M}{\rho RT}(\frac{3}{\pi\beta})^{\frac{1}{3}} f(\theta)dt \qquad \text{(S-16)}$$

Integrating equation (S-16):

$$V^{\frac{2}{3}} = V_0^{\frac{2}{3}} - \frac{4}{3}\pi D \Delta P \frac{M}{\rho RT} (\frac{3}{\pi\beta})^{\frac{1}{3}} f(\theta) t \quad \text{(S-17)}$$

Coupled with equation (S-1) and equation (S-6), the evaporation equation can be written into another form with specific surface area (S_0/V_0):

$$(\frac{V}{V_0})^{\frac{2}{3}} = 1 - \frac{4}{3}\pi D \Delta P \frac{M}{\rho RT} (\frac{\pi\beta}{3})(\frac{1}{2\pi(1-\cos\theta)})^2 f(\theta)(\frac{S_0}{V_0})^2 t \quad \text{(S-18)}$$

Collating the equation, then:

$$(\frac{V}{V_0})^{\frac{2}{3}} = 1 - D \Delta P \frac{M}{\rho RT} (\frac{2+\cos\theta}{9}) f(\theta)(\frac{S_0}{V_0})^2 t \quad \text{(S-19)}$$

Seen from equation (S-19), when droplet exposing in air, the decrease of relative volume of droplet (V/V0) mainly depends on the specific surface area of droplet (S0/V0). Therefore, the smaller droplet has higher evaporation ability due to its larger specific surface area.

The relative volume (V /V0) variation of different volumes of droplets (i.e., 0.2 μL, 1 μL, 5 μL) calculated by theoretical model above were shown in **Figure S3b**. The figure shown that, compared with 5 μL and 1 μL droplet, the relative volume of 0.2 μL droplet reduced more remarkably with the increase of time. After 2 minutes, the 0.2 μL droplet decreased to about 40% of initial volume, but the 1 μL and 5 μL droplet had remained about 80% and 90% of initial volume, respectively. The theoretical results agreed well with the experiments, and both of them indicated that the evaporation of small droplets (0.2 μL) cannot be ignored and induced the decrease of cell viability.

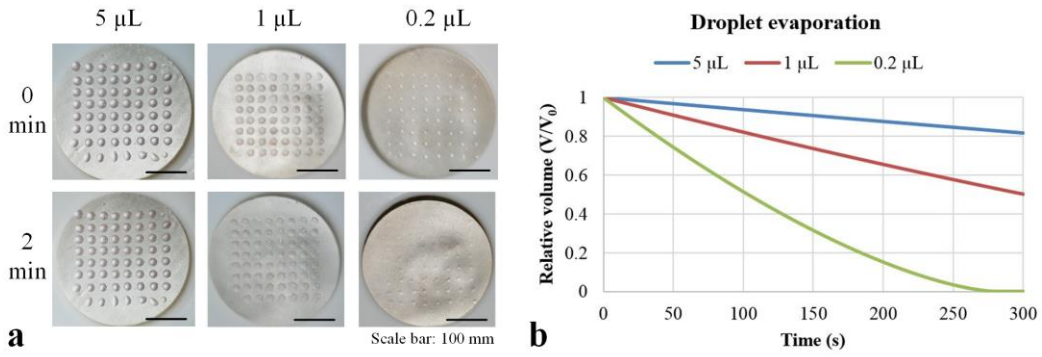

Figure S3 Droplets evaporation analysis. (a) Morphology of different volumes of droplets at initial and after 2 min; (b) Theoretical evaporation curve of different volumes of droplets.

References

1 Wowk, B. Thermodynamic aspects of vitrification. *Cryobiology* **60**, 11-22 (2010).

2 Boutron, P. Comparison with the theory of the kinetics and extent of ice crystallization and of the glass-forming tendency in aqueous cryoprotective solutions. *Cryobiology* **23**, 88-102 (1986).

3 Jin, T., Hong, J.-p., Zheng, H., Tang, K. & Gan, Z.-h. Measurement of boiling heat transfer coefficient in liquid nitrogen bath by inverse heat conduction method. *J. Zhejiang Univ. Sci. A* **10**, 691-696, (2009).

4 Jankowski, J. E. *Convective heat transfer model for determining quench recovery of high temperature superconducting YBCO in liquid nitrogen*, PhD diss., Massachusetts Institute of Technology, (2004).

5 Schönfeld, F., Graf, K.-H., Hardt, S. & Butt, H.-J. Evaporation dynamics of sessile liquid drops in still air with constant contact radius. *Int. J. Heat Mass Tran.* **51**, 3696-3699, (2008).

Movie S1 The freezing process of a 5 μL droplet hanging on the freezing film of non-contact vitrification device
